# Disseminated intravascular coagulation with increased fibrinolysis during the early phase of isolated traumatic brain injury

**DOI:** 10.1186/s13054-017-1808-9

**Published:** 2017-08-22

**Authors:** Takeshi Wada, Satoshi Gando, Kunihiko Maekaw, Kenichi Katabami, Hisako Sageshima, Mineji Hayakawa, Atsushi Sawamura

**Affiliations:** Division of Acute and Critical Care Medicine, Department of Anesthesiology and Critical Care Medicine, Hokkaido University Graduate School of Medicine, N15W7, Kita-ku, Sapporo, 060-8638 Japan

**Keywords:** Disseminated intravascular coagulation, Fibrinolysis, Fibrinogenolysis, Isolated traumatic brain injury, Outcome

## Abstract

**Background:**

There is evidence to demonstrate that the coagulopathy which occurs in patients with traumatic brain injury coincides with disseminated intravascular coagulation (DIC). We hypothesized that DIC with increased fibrinolysis during the early stage of isolated traumatic brain injury (iTBI) affects the outcome of the patients and that hypoperfusion contributes to hyperfibrinolysis in the DIC.

**Methods:**

This retrospective study included 92 patients with iTBI who were divided into DIC and non-DIC groups according to the Japanese Association Acute Medicine DIC scoring system. The DIC patients were subdivided into those with and without hyperfibrinolysis. The platelet counts and global markers of coagulation and fibrinolysis were measured. Systemic inflammatory response syndrome (SIRS), organ dysfunction (assessed by the Sequential Organ Failure Assessment score), tissue hypoperfusion (assessed by the lactate levels) and the transfusion volume were also evaluated. The outcome measure was all-cause hospital mortality.

**Results:**

DIC patients showed consumption coagulopathy, lower antithrombin levels and higher fibrin/fibrinogen degradation products (FDP) and D-dimer levels than non-DIC patients. All of the DIC patients developed SIRS accompanied by organ dysfunction and required higher blood transfusion volumes, leading to a worse outcome than non-DIC patients. These changes were more prominent in DIC with hyperfibrinolysis. A higher FDP/D-dimer ratio suggests that DIC belongs to the fibrinolytic phenotype and involves fibrin(ogen)olysis. The mean blood pressures of the patients with and without DIC on arrival were identical. Hypoperfusion and the lactate levels were not identified as independent predictors of hyperfibrinolysis.

**Conclusions:**

DIC, especially DIC with hyperfibrinolysis, affects the outcome of patients with iTBI. Low blood pressure-induced tissue hypoperfusion does not contribute to hyperfibrinolysis in this type of DIC.

**Electronic supplementary material:**

The online version of this article (doi:10.1186/s13054-017-1808-9) contains supplementary material, which is available to authorized users.

## Background

There is evidence to demonstrate that the coagulopathy which occurs in patients with traumatic brain injury coincides with the definition of disseminated intravascular coagulation (DIC) by the International Society on Thrombosis and Haemostasis (ISTH): the intravascular activation of coagulation with the loss of localization and damage to the microvasculature [1, 2]. Kaufman et al. [3] showed clear evidence of consumption coagulopathy and the formation of disseminated microvascular thrombi in the brain and other organs, particularly in the lungs. Similarly, many studies using special staining techniques have revealed intravascular thrombosis in the injured brain [4]. Intravascular microthrombosis is strongly linked to neuronal death and affects the outcome as a secondary cerebral insult following initial brain damage [5–7]. In addition, endothelial activation and injury that occurs in association with intravascular tissue factor and thrombin formation, and systemic inflammation have been observed in patients with traumatic brain injury, especially among the patients who showed worse outcomes [8, 9].

DIC is divided into fibrinolytic (hemorrhagic) and thrombotic phenotypes [10, 11]. DIC in the early phase of trauma belongs to the fibrinolytic phenotype and is associated with a poor prognosis [12, 13]. If it does not improve, the DIC then proceeds to the thrombotic phenotype [13]. Since the first report of DIC in traumatic brain injury, many studies have pointed out that extremely elevated levels of fibrin/fibrinogen degradation products (FDP) and D-dimer are associated with a poor outcome [14–17]. These results suggest that DIC with the fibrinolytic phenotype also affects the prognosis of patients with traumatic brain injury [18].

DIC with the fibrinolytic phenotype is defined by the coexistence of both DIC and pathological systemic fibrin(ogen)olysis [10, 11]. In the early phase of trauma and traumatic shock, shock-induced hypoperfusion releases tissue-type plasminogen activator (t-PA) from the endothelial Weibel–Palade bodies, resulting in systemic primary fibrin(ogen)olysis in addition to DIC-induced secondary fibrinolysis [1, 10, 12, 13]. In the clinical setting, hemorrhagic shock is rarely associated with isolated traumatic brain injury (iTBI). Although the relationship between early coagulopathy and hypoperfusion was implicated in iTBI, the participation of hypoperfusion in increased fibrin(ogen)olysis has not been elucidated [19]. In addition, a recent study suggested that participation of endogenous plasminogen activators is involved in the increased fibrinolysis after iTBI, which causes progressive intracerebral hemorrhage [20].

Based on these insights derived from previous studies, we tested the hypotheses that DIC associated with increased fibrinolysis affects the outcome of patients with iTBI and that hypoperfusion contributes to the increased fibrinolysis in this type of DIC.

## Methods

### Patients

Consecutive patients with traumatic brain injury who were admitted to the emergency department (ED) from June 2000 to March 2016 were eligible for inclusion in the present study. Patients with severe iTBI who were admitted to the ICU were included in the present study. Exclusion criteria were as follows: missing data, ≤ 8 years of age, cardiac arrest and resuscitation, severe injuries to other parts of the body and cervical spinal cord injury. Our Institutional Review Board approved this study and waived informed consent.

We retrospectively conducted a systematic review of the computer-based medical records of these patients to provide baseline data and DIC-related parameters. The platelet count, prothrombin time, prothrombin time ratio, fibrinogen, antithrombin, FDP, D-dimer and lactate levels were measured at four time points within 24 hours after admission to the ED: Time Point 01, immediately after to 4 hours after arrival at the ED; Time Point 02, 4–8 hours after arrival; Time Point 03, 8–16 hours after arrival; and Time Point 04, 16–24 hours after arrival. The day 0 data showed the worst maximal values (highest or lowest) of these four measurement time points. The day 0 data were used for classifying patients with or without DIC and for determining the phenotype.

### Definitions

Severe isolated traumatic brain injury (iTBI) was defined according to the Abbreviated Injury Scale (AIS) as follows: AIS of the head and neck ≥ 3 and AIS of other body parts ≤ 2. The Injury Severity Score (ISS) was used to evaluate the degree of the injury. In the present study, brain damage was defined as cerebral contusion with or without hematoma and subdural hematoma with cerebral contusion. The severity of the patient’s condition and organ dysfunction was assessed by the Acute Physiology and Chronic Health Evaluation II (APACHE II) score and the Sequential Organ Failure Assessment (SOFA) score [21], respectively. The systemic inflammatory response syndrome (SIRS) score was calculated according to the formula established in the American College of Chest Physicians/Society of Critical Care Medicine consensus conference [22]. DIC was diagnosed based on the Japanese Association for Acute Medicine (JAAM) DIC and the International Society on Thrombosis and Haemostasis (ISTH) overt DIC diagnosis criteria based on the day 0 data [1, 23]. DIC and overt DIC were diagnosed based on total scores of ≥ 4 and ≥ 5, respectively. We referred to the criteria of Asakura [11] for the definition of the DIC phenotype. Hyperfibrinolysis was defined as an FDP level ≥ 100 μg/ml, and the FDP/D-dimer ratio was used as a surrogate marker of fibrin(ogen)olysis [11]. Tissue hypoperfusion was defined as a blood lactate level ≥ 4 mmol/L based on the Surviving Sepsis Campaign Guidelines 2012 [24]. The outcome measure was the rate of all-cause hospital mortality.

### Statistical analyses

The results were shown as the median and interquartile range. The IBM SPSS software program (version 24.0 for MAC OSX; IBM Japan, Tokyo, Japan) was used for all of the analyses. The Kruskal–Wallis test was used for comparison among three groups, while the Mann–Whitney *U* test and the chi-square test were used for comparisons between two groups. The dependent and the independent variables were evaluated by logistic regression analysis (the backward stepwise method based on likelihood) and the odds ratios and 95% confidence intervals were shown. The prediction of hospital death was evaluated using the area under the receiver operating characteristic (ROC) curve (AUC). Survival curves during hospital stay were analyzed by the Kaplan–Meier method using a log-rank test for comparison. *p* < 0.05 was considered to indicate statistical significance.

## Results

### Demographic and clinical characteristics of the patients

**Fig. 1:**
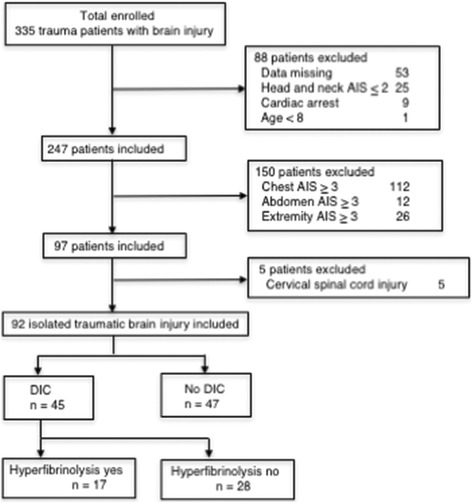
Flowchart of the patients’ selection process. DIC diagnosed based on the JAAM DIC scoring system. *AIS* Abbreviated Injury Scale, *DIC* disseminated intravascular coagulation

During the study period, a total of 335 trauma patients with brain injury presented to the ED. After the exclusion of ineligible patients and patients with incomplete data, patients with cervical spinal cord injury in whom the AIS of the head and neck was ≥ 3 were further excluded. Finally, 92 eligible patients with iTBI were identified. The patients were divided into the DIC (*n* = 45) and non-DIC (*n* = 47) groups based on the JAAM DIC scoring system (Fig. 1).

Table 1 presents the demographic data of the patients. Although the ISS and AIS were distributed evenly between the two groups, the APACHE II scores of the DIC patients were higher. All of the DIC patients developed SIRS with higher SOFA scores and required greater volume of blood transfusion, leading to a significantly worse outcome in comparison to the non-DIC patients. It is important to note that the mean blood pressures of the two groups at arrival to the ED were ≥ 90 mmHg and ﻿that only 5 patients required catecholamine, which suggests a low incidence of hemorrhagic shock in both groups of the patients. The use of tranexamic acid was evenly distributed between the two groups. No patients received an infusion or transfusion in the prehospital setting because emergency medical service personnel are not permitted perform transfusion and the infusion of Ringer’s lactate solution is only allowed for patients with a severe worsening of hypovolemic shock.

**Table 1:** Demographic and clinical characteristics of the whole study population

	No DIC	DIC	*p* value
	(47)	(45)	
Age (years)	49 (31–64)	56 (36–70)	0.261
Male sex	37 (78.7)	30 (66.7)	0.244
Time from call receipt to arrival at ED	31.0 (22.8–43.0)	31.5 (24.0–37.0)	0.799
JAAM DIC score	1 (1–2)	4 (4–6)	0.000
ISTH DIC score	1 (0–2)	3 (3–4)	0.000
ISTH DIC	0 (0)	7 (15.6)	0.000
Hyperfibrinolysis	0 (0)	17 (37.8)	0.000
APACHE II score	18 (11–27)	24.0 (18–31)	0.028
Injury Severity Score	24 (17–26)	24.0 (19–26)	0.330
Abbreviated Injury Scale			
Head and neck	4 (4–5)	4 (4–5)	0.154
Face	2 (2–3)	2 (2–2)	0.309
Chest	2 (1–2)	1 (1–1)	0.282
Abdomen	2 (2–2)	2 (2–2)	0.825
Extremity	2 (1–2)	2 (1–2)	0.934
External	1 (1–2)	1 (1–1)	0.281
SIRS score	3 (3–3)	4 (3–4)	0.000
SIRS	44 (93.6)	45 (100)	0.215
SOFA score	4 (3–5)	5 (4–7)	0.000
Mean blood pressure (mmHg)	90 (80–109)	91 (76–112)	0.821
Catecholamine (n,%)	1 (2.1)	4 (8.9)	0.198
Hypoperfusion	11 (23.4)	29 (64.4)	0.000
Lactate (mmol/L)	2.9 (2.1–4.0)	4.6 (3.6–7.0)	0.000
Transfusion			
Red blood cell concentrate (ml)	0 (0–0)	140 (0–390)	0.000
Fresh frozen plasma (ml)	0 (0–0)	0 (0–240)	0.000
Platelet concentrate (U)	0 (0–0)	0 (0–0)	0.995
Tranexamic acid (n,%)	5 (10.6)	13 (28.9)	0.025
Outcome death	5 (10.6)	13 (28.9)	0.025

Data presented as median (interquartile range) or *n* (%)

*DIC* disseminated intravascular coagulation, *ED* emergency department, *JAAM* Japanese Association for Acute Medicine, *ISTH* International Society on Thrombosis and Haemostasis, *APACHE* Acute Physiology and Chronic Health Evaluation, *SIRS* systemic inflammatory response syndrome, *SOFA* Sequential Organ Failure Assessment

### Serial changes in the platelet count and markers of coagulation and fibrinolysis

**Fig. 2:**
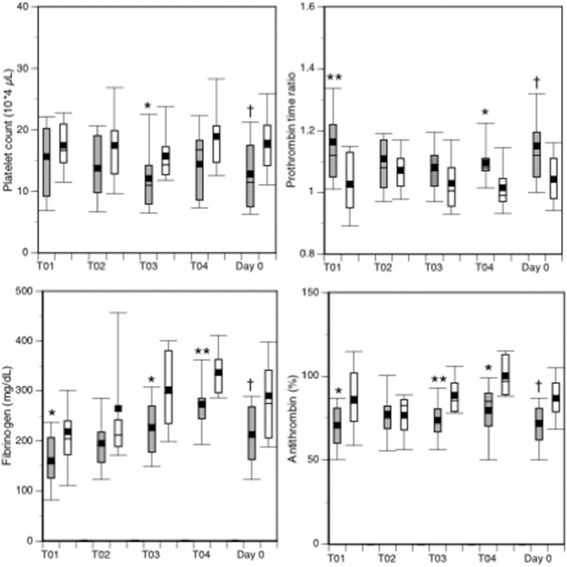
Box plots of platelet counts, prothrombin time ratios, and fibrinogen and antithrombin levels. *Dark boxes*, DIC patients; *white boxes*, non-DIC patients. *Horizontal bars* in the box indicate the median (middle) and interquartile ranges (upper 25%, lower 75%). *Black squares* in the box indicate the mean value. *Top and bottom bars* indicate the maximum and minimum values, respectively. *T01* Time Point 01 (immediately after to 4 hours after arrival at the ED), *T02* Time Point 02 (4–8 hours after arrival), *T03* Time Point 03 (8–16 hours after arrival), *T04* Time Point 04 (16–24 hours after arrival), *Day 0* worst maximal values (highest or lowest) of these four measurement time points. *p < 0.05, **p < 0.01, †*p* < 0.001 vs non-DIC patients

**Fig. 3:**
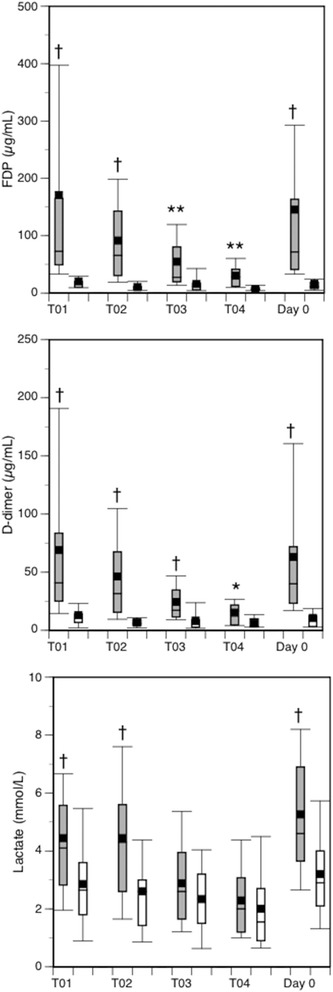
Box plots of FDP, D-dimer and lactate levels. *Dark boxes*, DIC patients; *white boxes*, non-DIC patients. *Horizontal bars* in the box indicate the median (middle) and interquartile ranges (upper 25%, lower 75%). *Black squares* in the box indicate the mean value. *Top and bottom bars* indicate the maximum and minimum values, respectively. *FDP* fibrin/fibrinogen degradation products, *T01* Time Point 01 (immediately after to 4 hours after arrival at the ED), *T02* Time Point 02 (4–8 hours after arrival), *T03* Time Point 03 (8–16 hours after arrival), *T04* Time Point 04 (16–24 hours after arrival), *Day 0* worst maximal values (highest or lowest) of these four measurement time points. *p < 0.05, **p < 0.01, †*p* < 0.001 vs non-DIC patients

**Fig. 4:**
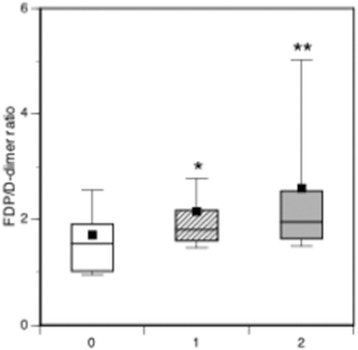
Box plots of the FDP/D-dimer ratios. *White box*, non-DIC; *hatched box*, DIC; *dark box*, DIC with hyperfibrinolysis. Kruskal–Wallis *p* = 0.002. *p = 0.002, **p = 0.006 vs non-DIC patients. *FDP* fibrin/fibrinogen degradation products

The DIC patients continuously showed significantly lower platelet counts, more prolonged prothrombin time ratios and lower levels of fibrinogen and antithrombin immediately after arrival at the ED (Time Point 01) until Time Point 04, and on day 0 in comparison to non-DIC patients (Fig. 2). The DIC patients also showed persistently higher levels of FDP and D-dimer in comparison to the non-DIC patients (Fig. 3). In addition, the FDP/D-dimer ratios of the DIC patients were significantly higher than those of the non-DIC patients (Fig. 4). These results suggest consumption coagulopathy, insufficient anticoagulation by antithrombin and increased fibrin(ogen)olysis in DIC patients. The numbers of patients at each time point are presented in Additional file 1: Table S1.

### Subgroup analyses of the DIC patients

The DIC patients were subdivided into those with (*n* = 17) and without (*n* = 28) hyperfibrinolysis based on their FDP levels (Table 2). The DIC patients’ fibrinolysis marker levels are presented in Table 3, which shows that the levels of FDP and D-dimer of the DIC patients with hyperfibrinolysis were extremely high in comparison to those without hyperfibrinolysis. The ISS of the two groups was identical; however, the patients with hyperfibrinolysis had higher DIC, APACHE II and SOFA scores and underwent transfusion with higher volumes of red blood cell concentrates in comparison to the patients without hyperfibrinolysis. The FDP/D-dimer ratios of the patients with hyperfibrinolysis were markedly higher than non-DIC patients (Fig. 4). Based on these results, DIC with hyperfibrinolysis is considered equivalent to DIC with the fibrinolytic phenotype, which is associated with fibrin(ogen)olysis, hemorrhage and organ dysfunction. DIC with hyperfibrinolysis was associated with a higher mortality rate (58.9%) in comparison to DIC without hyperfibrinolysis (10.7%).

**Table 2:** Demographic and clinical characteristics of the DIC patients

	DIC		
	Hyperfibrinolysis no	Hyperfibrinolysis yes	*p* value
	(*n* = 28)	(*n* = 17)	
Age (years)	45 (22–65)	66 (52–77)	0.008
Male sex	21 (75.0)	9 (52.9)	0.193
Brain injury	18 (64.2)	12 (70.6)	0.752
JAAM DIC score	4 (4–4.8)	5 (5–7)	0.000
ISTH DIC score	3 (3–3)	4 (3–5)	0.002
ISTH DIC	1 (3.6)	6 (35.3)	0.008
APACHE II score	20 (15–27)	28 (20–35)	0.040
Injury Severity Score	24 (18–26)	25 (21–28)	0.396
SIRS score	4 (3–4)	4 (3–4)	0.957
SOFA score	5 (4–6.8)	6 (5–7.5)	0.083
Transfusion			
Red blood cell concentrate (ml)	0 (0–192)	240 (140–540)	0.008
Fresh frozen plasma (ml)	0 (0–150)	120 (0–384)	0.127
Platelet concentrate (U)	0 (0–0)	0 (0–3.8)	0.118
Outcome death	3 (10.7)	10 (58.9)	0.001

Data presented as median (interquartile range) or *n* (%)

*DIC* disseminated intravascular coagulation, *JAAM* Japanese Association for Acute Medicine, *ISTH* International Society on Thrombosis and Haemostasis, *APACHE* Acute Physiology and Chronic Health Evaluation, *SIRS* systemic inflammatory response syndrome, *SOFA* Sequential Organ Failure Assessment

**Table 3:** Markers of fibrinolysis and hypoperfusion in the DIC patients with and without hyperfibrinolysis

	Hyperfibrinolysis no	Hyperfibrinolysis yes	*p* value
	(*n* = 28)	(*n* = 17)	
FDP (μg/ml)	48.8 (36.0–64.5)	198.0 (141.0–324.3)	0.000
D-dimer (μg/ml)	26.7 (18.8–38.9)	91.5 (69.0–174.2)	0.000
Mean blood pressure	91 (78–103)	106 (66–116)	0.699
Hypoperfusion	15 (53.6)	14 (82.4)	0.111
Lactate (mmol/L)	4.1 (3.0–5.6)	11.3 (8.5–15.6)	0.006

Data presented as median (interquartile range) or *n* (%)

*FDP* fibrin/fibrinogen degradation products

### Analyses of the outcomes and the predictors of hyperfibrinolysis

**Fig. 5:**
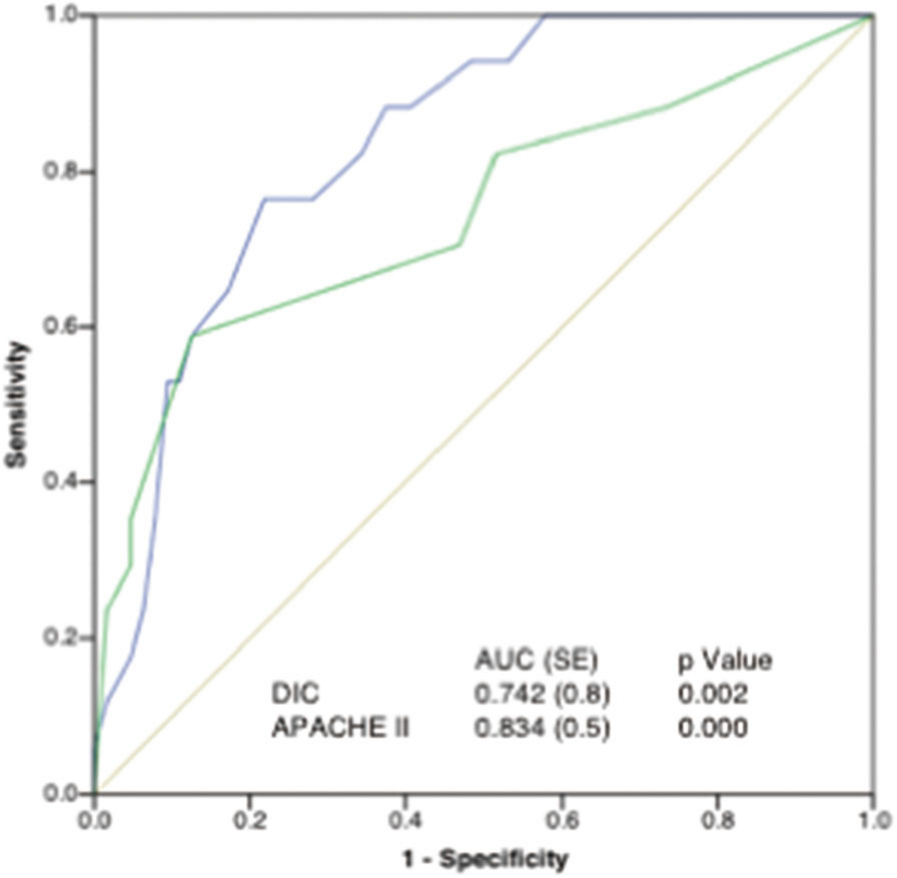
ROC curves of the DIC scores (*green line*) and APACHE II score (*blue line*) for predicting hospital death. *APACHE* Acute Physiology and Chronic Health Evaluation, *AUC* area under the receiver operating characteristic curve, *DIC* disseminated intravascular coagulation, *SE* standard error (Color figure online)

**Fig. 6:**
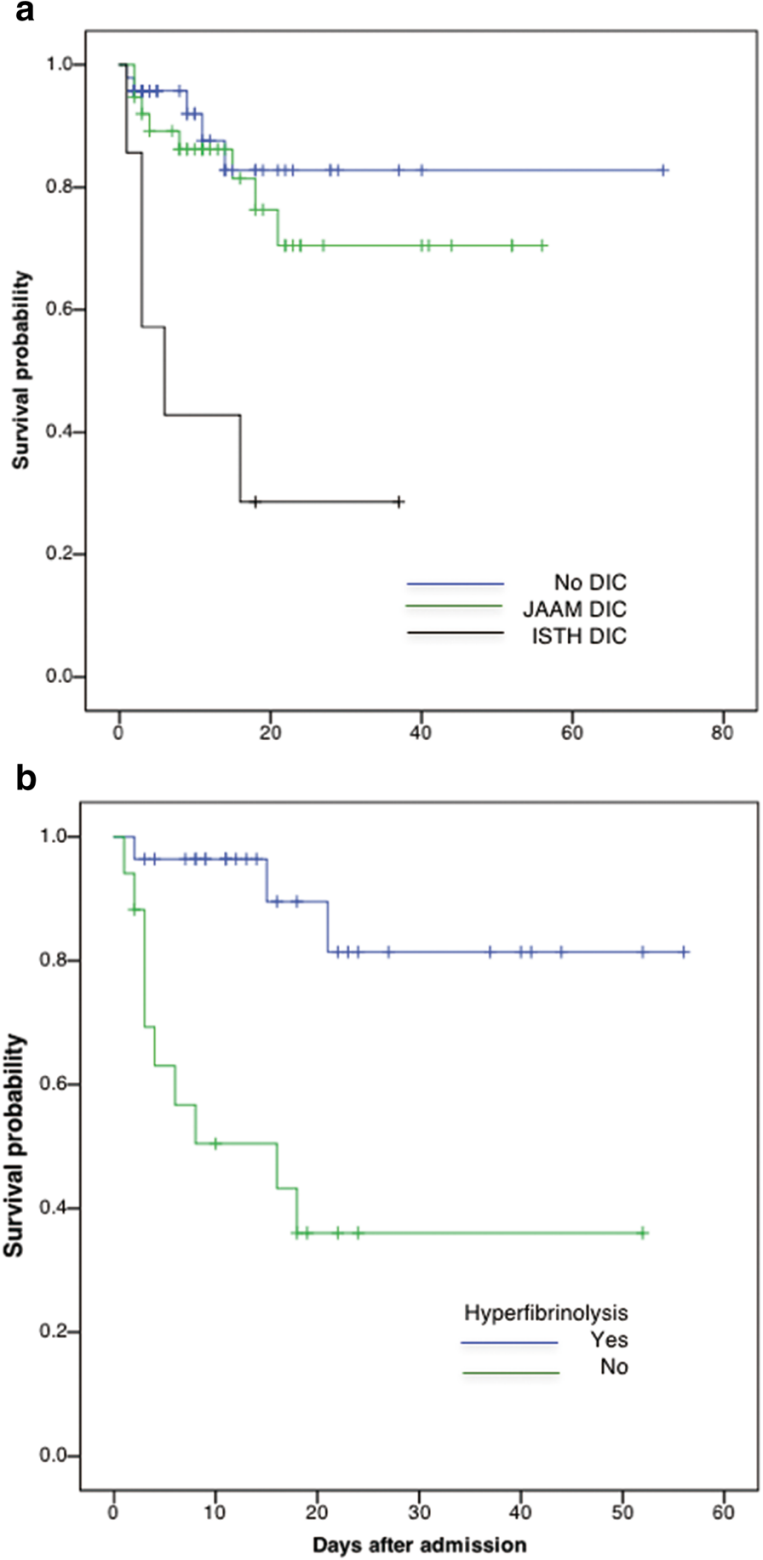
Kaplan–Meier curves showing the association between DIC (**a**) or DIC with hyperfibrinolysis (**b**) and hospital mortality. Log-rank *p* = 0.001 (**a**), *p* = 0.001 (**b**). *DIC* disseminated intravascular coagulation, *ISTH* International Society on Thrombosis and Haemostasis, *JAAM* Japanese Association for Acute Medicine (Color figure online)

Stepwise logistic regression analyses confirmed that the DIC and APACHE II scores are independent predictors of patients’ death (Table 4). The ROC curves further showed the significant discriminative performance of the DIC and APACHE II scores for predicting death in iTBI patients (Fig. 5). These results are important because the DIC score showed good discriminative power for predicting the outcome in comparison to the APACHE II score, which is known as a superior predictor of death in various kinds of critical illness. The Kaplan–Meier curves showed that DIC, especially DIC diagnosed by the ISTH scoring system and DIC with hyperfibrinolysis, significantly affected the survival rate of patients with iTBI (Fig. 6). Table 5 shows that the DIC score and the existence of hyperfibrinolysis further influenced the death in DIC patients.

**Table 4:** Results of logistic regression analysis of variables predicting the outcome (death) in the whole study population

	Enter method			Stepwise method		
	Odds ratio	*p* value	95% CI	Adjusted odds ratio	*p* value	95% CI
Age	1.022	0.230	0.986–1.058			
Sex	3.907	0.174	0.548–27.856			
JAAM DIC score	1.717	0.028	1.059–2.784	1.589	0.011	1.111–2.275
APACHE II score	1.219	0.006	1.059–1.402	1.194	0.001	1.075–1.327
Injury Severity Score	0.973	0.483	0.900–1.051			
SIRS score	1.486	0.614	0.319–6.910			
SOFA score	1.002	0.992	0.694–1.446			

Dependent variables: age, sex, JAAM DIC score, APACHE II score, Injury Severity Score, SIRS score, SOFA score. Results of the final step of the stepwise analyses are shown

*CI* confidence interval, *JAAM* Japanese Association for Acute Medicine, *DIC* disseminated intravascular coagulation, *APACHE* Acute Physiology and Chronic Health Evaluation, *SIRS* systemic inflammatory response syndrome, *SOFA* Sequential Organ Failure Assessment

**Table 5:** Results of logistic regression analyses of variables predicting the outcomes (death and hyperfibrinolysis) in DIC patients

	Enter method			Stepwise method		
	Odds ratio	*p* value	95% CI	Adjusted odds ratio	*p* value	95% CI
Death						
Age	1.008	0.703	0.966–1.052			
Sex	0.992	0.993	0.166–5.907			
JAAM DIC score	1.786	0.057	0.983–3.243	1.742	0.054	0.990–3.067
Hyperfibrinolysis	5.755	0.079	0.816–40.574	6.861	0.022	1.318–35.709
Hyperfibrinolysis						
Age	1.053	0.021	1.008–1.101	1.042	0.028	1.004–1.080
Sex	0.295	0.150	0.056–1.556			
Brain damage	0.481	0.420	0.081–2.846			
Hypoperfusion	2.423	0.430	0.269–21.85			
Lactate	1.181	0.393	0.806–1.728	1.335	0.058	0.991–1.798

Dependent variables: age, sex, JAAM DIC score, hyperfibrinolysis. Results of the final step of the stepwise analyses are shown

*CI* confidence interval, *JAAM* Japanese Association for Acute Medicine, *DIC* disseminated intravascular coagulation

Despite the fact that the two groups showed the same mean blood pressures on arrival at the ED, the DIC patients, especially those with hyperfibrinolysis, showed a higher prevalence of hypoperfusion and significantly higher lactate levels (Fig. 3, Tables 1 and 3). However, the lactate levels were not found to be an independent predictor of hyperfibrinolysis by the stepwise logistic regression analyses (odds ratio, 1.335; 95% confidence interval, 0.991–1.798; *p* = 0.058) (Table 5). In addition, neither hypoperfusion nor brain damage predicted hyperfibrinolysis in DIC patients.

## Discussion

The present study demonstrated that DIC—especially DIC with hyperfibrinolysis—during the early phase of iTBI is accompanied by systemic inflammation, organ dysfunction and greater need for transfusion. Furthermore, DIC significantly affects patient outcomes, which coincides with the trauma of the other body parts. Hemorrhagic shock is rare in iTBI patients and the tissue hypoperfusion evaluated by lactate levels did not contribute to hyperfibrinolysis in DIC patients with iTBI. The higher FDP/D-dimer ratios implicate the fibrin(ogen)olysis in this type of DIC.

Previous studies have separately discussed the roles of DIC and increased markers of fibrinolysis in the outcome of patients with traumatic brain injury or iTBI. DIC-induced microvascular thrombosis is closely linked to the area of ischemic changes and neuronal death in the injured brain [4, 5, 7]. Systemic microthrombi in vital organs such as the lungs, liver, kidneys, intestines and pituitary glands give rise to organ dysfunction and result in a worse outcome [3, 5, 9, 15]. Other studies have shown that DIC increases the FDP and D-dimer levels, which are independent predictors of progressive hemorrhage and a poor outcome in patients with traumatic brain injury [15–17]. The results of the present study unify DIC and increased FDP and D-dimer levels as one concept of DIC with hyperfibrinolysis, which leads to SIRS, organ dysfunction, a greater need for transfusion and worse outcomes in patients with iTBI.

van der Sande et al. [14] demonstrated that DIC with a fatal outcome was associated with extremely high FDP levels and assumed the coexistence of an underlying process that differed from DIC, namely primary fibrin(ogen)olysis due to an unknown cause. In the present study, higher FDP/D-dimer ratios in DIC—especially DIC with hyperfibrinolysis—implicated the existence of fibrin(ogen)olysis and suggested that the DIC belongs to the fibrinolytic phenotype [10, 11]. Another study showed a close relationship between DIC with fibrinolysis and delayed and recurrent intracranial hematomas in patients with head injury [25]. The same group demonstrated that DIC with fibrinolytic syndrome increased the mortality rate by > 4 times that of patients without DIC [18]. These studies support our hypothesis and results, which indicates that DIC with hyperfibrinolysis (the fibrinolytic phenotype) solely affects the outcome of patients with iTBI. The present study further suggests that DIC with the fibrinolytic phenotype in iTBI equally influences the patient’s outcome similar to DIC with the fibrinolytic phenotype in patients with extracranial trauma [2, 12, 13].

The consumption coagulopathy observed in the present study indicates that the increases in the levels of FDP and D-dimer are partly due to DIC-induced secondary fibrinolysis. Fibrin(ogen)olysis, which is thought to be present based on the higher FDP/D-dimer ratios, suggests that a condition other than iTBI increases the levels of the two markers. Thus, the involvement of coexisting condition(s) in hyperfibrinolysis was investigated. The present study failed to show a difference in the mean blood pressures on arrival at the ED between the DIC and non-DIC groups or DIC with and without hyperfibrinolysis. This means that the mechanism underlying the development of hyperfibrinolysis due to the shock-induced release of t-PA from the endothelial cells is unlikely. Although apparent shock was not observed, the higher lactate levels in DIC suggest the role of tissue hypoperfusion in the mechanisms of hyperfibrinolysis. However, the logistic regression analyses clearly demonstrated that tissue hypoperfusion was not a cause of hyperfibrinolysis in patients with iTBI-associated DIC. These results suggest that the lactate levels are not increased due to low blood pressure-related hypoperfusion and that they occurred due to DIC-induced low flow-related hypoperfusion in the damaged brain tissues [19, 26, 27].

Another explanation of hyperfibrinolysis in iTBI is that traumatic brain injury induces the release of t-PA from injured brain tissues [2]. Localized fibrinolytic activity and the expression of t-PA in the brain have long been reported to occur [28–31]. A recent study indicated that endogenous t-PA and urokinase-type plasminogen activator (u-PA) increase the lysis of plasma clots and contribute to intracerebral hemorrhage after traumatic brain injury [20]. Based on these studies, we investigated the relationship between brain damage and hyperfibrinolysis. However, the logistic regression analysis did not show brain damage to be the main cause of hyperfibrinolysis. A more detailed study should be performed in order to determine how the areas of brain damage are associated with the increases in FDP and D-dimer levels.

Lastly, the present study clearly demonstrated that DIC is associated with SIRS, organ dysfunction and the need for greater transfusion volumes, especially in patients with hyperfibrinolysis, leading to a worse outcome in patients with iTBI. The ROC curve suggests that the DIC score could be used for predicting the outcome of patients with iTBI. It is important to note that the possibility of survival in DIC with hyperfibrinolysis was < 40%. These results indicate the need to treat DIC as well as hyperfibrinolysis during the early stage of iTBI. The administration of tranexamic acid within 3 hours of iTBI may be a promising treatment; however, the late application of this drug might increase intracerebral hemorrhage by potentiating u-PA-mediated plasminogen activation. [32, 33].

The present study was associated with some limitations. This was a single-center retrospective study that involved a relatively small number of patients and was limited by incomplete data. We also acknowledge that the relatively small sample size in the present study negatively impacts the robustness of the logistic regression analyses. The measured markers were collected systemically and not locally from the brain. The molecular markers of fibrinolysis were not measured, nor were the relationships between increased fibrinolysis and intracerebral hemorrhage. It is necessary to elucidate how the release of t-PA from the injured brain tissues is involved in hyperfibrinolysis. Finally, over the long years of the study period the patients have changed treatments, which may act as confounders to the data.

## Conclusions

Patients with DIC during the early stage of iTBI showed lower platelet counts, consumption coagulopathy and insufficient coagulation control by antithrombin. Highly elevated FDP and D-dimer levels and increased FDP/D-dimer ratios suggest that DIC belongs to the fibrinolytic phenotype, which involves fibrin(ogen)olysis. DIC was associated with SIRS, organ dysfunction and need for greater transfusion volumes, especially in patients with hyperfibrinolysis, leading to worse outcomes in patients with iTBI. The lactate levels and the prevalence of hypoperfusion were increased in DIC patients. However, hemorrhagic shock was not observed and neither lactate levels nor hypoperfusion contributed to hyperfibrinolysis in DIC patients. The results failed to prove that damaged brain tissue contributes to hyperfibrinolysis; this mechanism should be investigated in a more detailed study in the future. To improve the outcome of the patients with iTBI, the control of both DIC and increased fibrinolysis will be necessary.

## Additional file

Additional file 1: Table S1. (XLSX 23 kb)

## Abbreviations

AIS: Abbreviated Injury Scale
APACHE: Acute Physiology and Chronic Health Evaluation
AUC: Area under the receiver operating characteristic curve
DIC: Disseminated intravascular coagulation
ED: Emergency department
FDP: Fibrin/fibrinogen degradation products
ISS: Injury Severity Score
ISTH: International Society on Thrombosis and Haemotasis
iTBI: Isolated traumatic brain injury
JAAM: Japanese Association for Acute Medicine
ROC: Receiver operating characteristic
SIRS: Systemic inflammatory response syndrome
SOFA: Sequential Organ Failure Assessment
t-PA: Tissue-type plasminogen activator
u-PA: Urokinase-type plasminogen activator
